# Through the Eyes of Uncertainty: Giant Cell Arteritis and Lyme Neuroborreliosis in a Story of Vision Loss

**DOI:** 10.7759/cureus.53623

**Published:** 2024-02-05

**Authors:** Leo Wan, Audrey Yan, Erin Reese, John Thomas, Mark Kissinger

**Affiliations:** 1 College of Medicine, West Virginia School of Osteopathic Medicine, Lewisburg, USA; 2 Family Medicine, Trinity Health System, Steubenville, USA

**Keywords:** giant cell arteritis (gca), internal medicine and rheumatology, vision changes, lyme's disease, lyme borreliosis, ophthalmology

## Abstract

Acute vision loss is a prevalent clinical manifestation associated with a broad spectrum of differential diagnoses, encompassing demyelinating diseases, neoplastic processes, autoimmune disorders, and infectious conditions. A rare but noteworthy infectious etiology contributing to acute vision loss is neurological Lyme disease (Lyme neuroborreliosis)-induced optic neuritis. Lyme disease, a vector-borne illness caused by the spirochete* Borrelia burgdorferi*, has the potential to affect multiple physiological systems and unfolds in three distinct stages. Another significant contributor to acute vision loss is giant cell arteritis, an autoimmune vasculitis that commonly affects large- and medium-sized vessels, including the temporal and ophthalmic arteries. This relatively common condition may manifest with symptoms, such as jaw claudication, headaches, and visual disturbances. The precise identification of the underlying cause of acute visual loss is of utmost importance for physicians, as it is instrumental in averting undesirable complications. An 80-year-old female presents to the emergency room with a sudden onset of blurry vision of the left eye, right-sided weakness, dysarthria, jaw pain, headache, and left facial droop. Following consultations with rheumatology and ophthalmology specialists, giant cell arteritis emerged as a primary consideration in the differential diagnosis for the observed vision loss. Subsequently, a temporal artery biopsy was conducted, definitively confirming the diagnosis of giant cell arteritis. Considering the patient's residence in an area endemic to Lyme disease, a Lyme immunoglobulin G (IgG) titer was ordered. The results returned positive, suggesting the presence of Lyme neuroborreliosis.

## Introduction

Acute vision loss presents as a common clinical manifestation with diverse underlying causes, including demyelinating diseases, neoplastic processes, autoimmune disorders, and infectious conditions. A meticulous and comprehensive initial patient history is crucial for accurate diagnosis and treatment, particularly when exploring rarer conditions, in order to prevent permanent vision loss. One autoimmune cause of sudden vision loss is giant cell arteritis (GCA), a persistent granulomatous vasculitis affecting large- and medium-sized vessels. Predominantly seen in individuals aged 50 and above, GCA's prevalence increases with age, reaching its peak between 70 and 79 years. Research suggests that the lifetime likelihood of developing GCA in the United States is approximately 1% among women and 0.5% among men [1]. GCA commonly affects the aorta and the branches of the carotid arteries, both intra- and extracranial, including the temporal and ophthalmic arteries. This results in symptoms, such as visual disturbances, headaches, and jaw claudication. Over time, GCA can lead to serious consequences, such as ischemia in afferent and/or efferent visual pathways, resulting in vision loss or diplopia. Approximately 20% of individuals with GCA experience vision loss, with arteritic anterior ischemic optic neuropathy (AAION) being the most prevalent cause, affecting 79-91% of these patients [1]. Ischemia occurs at the head of the optic nerve with structural crowing of the nerve fibers, impairing perfusion and leading to optic disc edema [2].

One infectious cause of acute vision loss is Lyme-induced optic neuritis. Lyme disease, caused by the spirochete *Borrelia burgdorferi*, is the most common vector-borne ailment in the United States, with its highest endemicity observed in the Northwest, Northeast, and a substantial portion of the North Central region. The disease typically presents with flu-like symptoms and a distinctive erythematous “bulls-eye” rash, known as erythema migrans, appearing seven to 14 days after a tick bite. As the disease progresses, individuals may present with a spectrum of manifestations, encompassing cardiac complications, such as carditis and atrioventricular block, neurologic sequelae including encephalitis and cranial nerve palsy, and musculoskeletal issues manifested through arthralgia and arthritis [2]. Although relatively rare, Lyme disease can also lead to optic neuritis, an optic nerve inflammation resulting in eye pain and blurry vision. This occurrence represents approximately 1% of systemic Lyme disease cases but warrants consideration in the differential diagnosis of acute vision loss [3].

In some cases, Lyme disease and GCA have been misdiagnosed interchangeably due to overlapping symptoms, such as blurry vision, headache, and optic disc edema [4,5]. We would like to highlight a specific case involving acute mononuclear vision loss, wherein the patient tested positive for both GCA and Lyme disease.

## Case presentation

An 80-year-old female with a medical history of hypertension and hypothyroidism presented to the emergency department in Pennsylvania with a sudden onset of complex neurological symptoms. These included left eye blurry vision (20/25 OD, 25/200 OS), left jaw pain, headache with temporal artery tenderness, transient left facial palsy, right-sided weakness, and dysarthria. Due to the multifaceted clinical presentation, a comprehensive diagnostic workup was conducted by Neurology, including multi-plane MRI sequences of the brain and orbits, and computed tomography angiography (CTA) of the head and neck to rule out a transient ischemic attack (TIA). Results from the diagnostic workup revealed no hemodynamically limiting stenosis, dissection, or large vessel occlusion, as well as no brain mass, acute infarction, or hemorrhage on CTA of the neck and head. Carotid Doppler indicated less than 50% stenosis bilaterally. The lipid panel showed triglyceride 56, cholesterol 134, low-density lipoprotein (LDL) 68, high-density lipoprotein (HDL) 55, and very low-density lipoprotein (VLDL) 11. A transthoracic echocardiogram was within normal limits. While the patient experienced complete resolution of facial palsy, weakness, and dysarthria with no evidence of acute stroke, the symptoms were concerning for a transient ischemic attack. As a result, the patient was recommended to continue acetylsalicylic acid (ASA) 81 mg daily and Brilinta for 21 days from a stroke perspective, with an antiplatelet plan to be determined after 21 days by cardiology and the primary care physician. 

**Figure 1:**
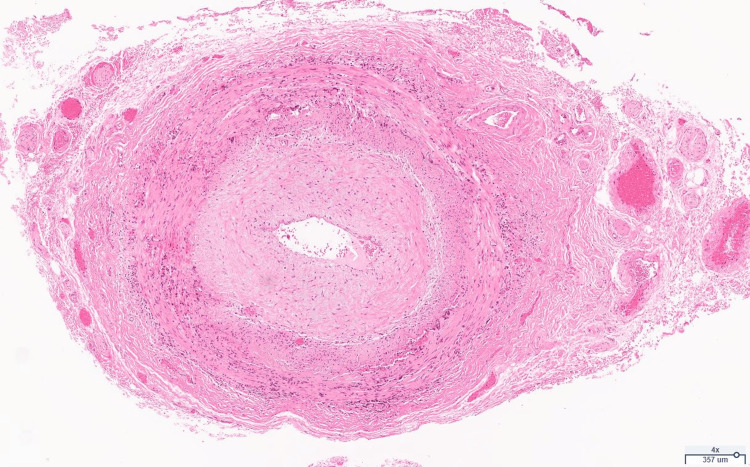
Temporal artery biopsy, giant cell arteritis, low-power magnification. Note the presence of a focal inflammatory infiltrate and intimal hyperplasia.

**Figure 2:**
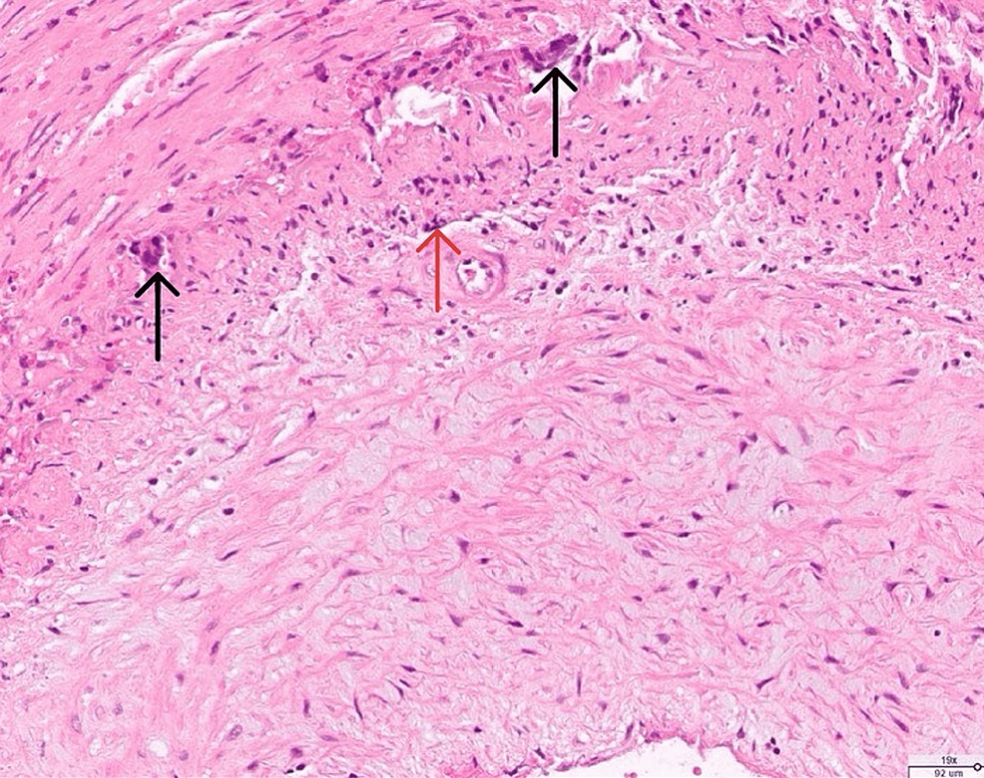
Temporal artery biopsy, giant cell arteritis, high-power magnification. Note the presence of multinucleated giant cells (black arrowhead) and mononuclear cell infiltration (red arrowhead).

Consultations were initiated with both rheumatology and ophthalmology. Laboratory testing included antinuclear antibody (ANA) at 1:160, rapid plasma reagin (RPR) negative, antineutrophil cytoplasmic antibody (ANCA) negative, anti-Sjögren's syndrome A (SSA)/anti-Sjögren's syndrome A (SSB) negative, *Bartonella* negative, and toxoplasmosis negative. An optical coherence tomography (OCT) of the optic nerve showed an optic disc edema. GCA emerged as a prominent consideration, prompting a temporal artery biopsy. The biopsy results confirmed the diagnosis, revealing a focal inflammatory infiltrate, intimal hyperplasia (Figure 1), multinucleated giant cells, and mononuclear cell infiltration (Figure 2).

High-dose 40 mg corticosteroids were initiated, resulting in a gradual improvement in the patient's unilateral vision loss. Rheumatology recommended the initiation of tocilizumab for GCA, pending the exclusion of active infections. A comprehensive series of infectious disease testing panels, including Lyme titers, were initiated. Surprisingly, the Lyme IgG returned positive in five bands on the Western blot, suggesting a diagnosis of Lyme neuroborreliosis. The patient declined lumbar puncture for definitive diagnosis but was recommended treatment by both rheumatology and ophthalmology. A treatment regimen for Lyme neuroborreliosis was implemented, involving IV ceftriaxone at 2 grams daily for 14 days, followed by 100 mg doxycycline twice a day orally for an additional 14 days. Upon discharge, the patient's vision in the left eye remained reduced but was improving and stable (20/25 OD, 20/100 OS).

## Discussion

Lyme disease is one of the most prevalent arthropod-borne illnesses in the Northern Hemisphere, and the number of reported cases in the United States has increased from 7,943 in 1990 to 18,000 in 2020 [6-7]. It has many clinical manifestations and affects the body's multisystem. Acute vision loss can be a rare manifestation of Lyme disease. In the typical progression of Lyme disease, ocular manifestations emerge during the second or third stage. These manifestations can vary widely and may include conditions, such as conjunctivitis, cranial nerve palsies (II, III, IV, and VII), keratitis, uveitis, vascular occlusion, and optic neuritis. Notably, optic neuritis is infrequent in Lyme borreliosis, often leading to its omission from consideration in the initial differential diagnosis for patients with acute vision loss [8]. Untreated Lyme neuroborreliosis may lead to delayed neurological manifestations, such as encephalomyelitis, chronic meningitis, and cerebral vasculitis, manifesting months or even years post-infection [6].

The current guidelines set forth by the Centers for Disease Control and Prevention (CDC) advocate for an initial, sensitive screening using enzyme-linked immunosorbent assay (ELISA) to detect IgG and IgM antibodies, followed by a confirmatory western immunoblot for Lyme disease. However, diagnosing the early stages of Lyme neuroborreliosis necessitates a more direct approach involving detecting the causative agent in cerebrospinal fluid (CSF). As such, it is imperative for patients exhibiting symptoms indicative of Lyme neuroborreliosis to undergo thorough CSF and serum antibody testing for Borrelia burgdorferi. In addition, physicians should exercise caution in interpreting results, acknowledging that numerous cases of Lyme neuroborreliosis may go undiagnosed [6].

GCA is another common cause of acute vision loss. Diagnosis relies on the criteria developed by the American College of Rheumatology, which includes the following factors: onset age of 50 years or older (absolute requirement); the presence of new headaches, morning shoulder or neck stiffness, jaw or tongue claudication, or sudden vision loss; temporal artery abnormalities during examination including tenderness upon palpation and reduced pulsation; bilateral axillary involvement on imaging; an erythrocyte sedimentation rate equal to or exceeding 50 mm/h or C-reactive protein equal to or exceeding 10 mg/liter; a positive temporal artery biopsy or temporal artery halo sign on ultrasound; and fluorodeoxyglucose-positron emission tomography activity throughout the aorta [4].

The current recommended therapeutic approach for Lyme neuroborreliosis involves two to three weeks of intravenous ceftriaxone at a dosage of 2 grams daily [6]. Although some reports suggest that a three-week course of 200 mg doxycycline by mouth may be an equivalent treatment option, intravenous ceftriaxone remains the primary therapy for neurological involvement in Lyme disease due to its superior central nervous system penetration [4].

By contrast, the recommended treatment strategy for GCA consists of high-dose oral prednisone administered daily at a range of 40-60 mg. A gradual steroid taper can be initiated following the resolution of laboratory abnormalities and symptoms. In cases involving visual impairment, pulsatile intravenous methylprednisolone (500-1000 mg) is recommended initially, followed by the transition to oral steroids [1]. For individuals unsuitable for prolonged steroid use, alternative therapeutic options, such as tocilizumab, methotrexate, abatacept, ustekinumab, cyclophosphamide, azathioprine, and anti-tumor necrosis factor agents, present viable alternatives [1].

The patient was put on high-dose corticosteroids (40 mg) with tapering for GCA, along with a treatment regimen for Lyme neuroborreliosis, IV ceftriaxone 2 grams daily for 14 days, followed by 100 mg doxycycline twice a day by mouth for additional 14 days.

The patient exhibited distinctive clinical manifestations in the presented case, including unilateral facial paralysis, headache, jaw pain, and unilateral visual defects. Comprehensive diagnostic assessments were conducted to eliminate ischemic events, such as stroke and transient ischemic attack from consideration. However, due to the patient's refusal to undergo lumbar puncture for CSF analysis, a definitive determination regarding the primary etiology of the visual defects remains uncertain. The observed symptomatology raises questions regarding whether the visual impairments are primarily attributed to GCA or if Lyme neuroborreliosis is a contributory factor, given the potential overlap of symptoms. It is less likely that the patient's ocular symptoms were attributable to Lyme neuroborreliosis, particularly considering the improvement observed after a high dose of steroids. However, the possibility cannot be entirely ruled out due to the patient's decision to decline a lumbar puncture. Interestingly, there has been a case report where GCA was suspected to be induced by Lyme disease, where the temporal artery biopsy under Warthin-Stary silver stain shows a Borrelia spirochete within the giant cell [4]. However, spirochete was absent in this patient’s biopsy. Given the exceedingly rare presentation observed, further research is imperative to comprehensively elucidate the underlying pathological processes and explore the potential of Lyme disease to induce GCA.

Within the literature, numerous case reports recount instances of neuroborreliosis initially misdiagnosed and treated as GCA due to overlapping symptoms. However, a closer examination of CSF analysis ultimately revealed the accurate diagnosis of Lyme neuroborreliosis [5].

## Conclusions

Lyme neuroborreliosis-induced optic neuritis represents a rare manifestation of acute vision loss. Nonetheless, in the absence of timely intervention, patients may encounter more severe complications as the disease advances. Consequently, healthcare providers in a Lyme endemic area must include Lyme disease in their list of differential diagnoses when assessing patients with acute vision loss/disturbance, as there exists a risk of it being either undiagnosed or overshadowed by other presenting illnesses with analogous symptoms, such as GCA.
